# Trends in diabetes prevalence, awareness, treatment, and control in French-speaking Switzerland

**DOI:** 10.1038/s41598-024-54856-6

**Published:** 2024-02-28

**Authors:** Ariane Pauli, Carlos de Mestral, Pedro Marques-Vidal

**Affiliations:** 1 Faculty of Biology and Medicine, University of Lausanne, Lausanne, Switzerland; 2 Department of Primary Care Medicine, Geneva University Hospital, Population Epidemiology Unit, Geneva, Switzerland; 3 Department of Medicine, Internal Medicine, Lausanne University Hospital and University of Lausanne, Rue du Bugnon 46, 1011 Lausanne, Switzerland

**Keywords:** Diabetes, Antidiabetic treatment, Glycaemic control, Switzerland, Epidemiology, Diabetes

## Abstract

Diabetes is increasing in Switzerland, but whether its management has improved is unknown. We aimed to assess diabetes prevalence, diagnosis, treatment, and control in French-speaking Switzerland. Our study used cross-sectional data for years 2005–2019 from a population-based study in Geneva, Switzerland. Overall prevalence (self-reported diagnosis and/or fasting plasma glucose level ≥ 7 mmol/L), diagnosed, treated (among diagnosed participants) and controlled diabetes (defined as a fasting plasma glucose FPG < 6.7 mmol/L among treated participants) were calculated for periods 2005–9, 2010–4 and 2015–9. Data from 12,348 participants (mean age ± standard deviation: 48.6 ± 13.5 years, 51.7% women) was used. Between 2005–9 and 2015–9, overall prevalence and frequency of diagnosed diabetes decreased (from 8.7 to 6.2% and from 7.0 to 5.2%, respectively). Among participants diagnosed with diabetes, treatment and control rates did not change from 44.1 to 51.9%, *p* = 0.251 and from 30.2 to 34.0%, *p* = 0.830, respectively. A trend towards higher treatment of participants with diabetes was found after multivariable adjustment, while no changes were found for overall prevalence, diagnosis, nor control. Among antidiabetic drugs, percentage of combinations increased from 12 to 23%; percentage of sulfonylureas and biguanides decreased from 15 to 6% and from 63 to 54%, respectively, while no trend was found for insulin. After multivariable analysis, women with diabetes were less likely to be treated but more likely to be controlled, the opposite association being found for obesity. In conclusion, in Canton Geneva, antidiabetic combination therapy is gaining importance, but only half of participants diagnosed with diabetes are treated, and glycaemic control remains poor.

## Introduction

Worldwide diabetes prevalence and incidence have increased significantly from 1990 to 2017. Population aging, as well as the rise in overweight and obesity, linked to suboptimal nutrition and sedentary lifestyles, has contributed to this tendency^1,2^. Diabetes, as one of the major causes of mortality and morbidity around the world, represents a constantly increasing global health burden^3^. However, control of diabetes and cardiovascular disease in Europe, including Switzerland, remains poor^4,5^.

National health surveys in Switzerland indicate only a minor increase of diabetes prevalence over the last years^6^. However, the probability of discovering an undiagnosed case of diabetes in the Swiss population, particularly among men, appears to be rising^7^. In addition, disparities in diabetes prevalence among various socioeconomic and cardiovascular risk groups in the Geneva population have increased over 13 years. Compared with adults with a higher socioeconomic background,disadvantaged adults were less aware of their diabetic condition^8^.

Swiss and international guidelines recommend metformin as first-line drug treatment for type 2 diabetes (T2DM), unless not well tolerated or contraindicated^9,10^. If necessary, other medications can be added to metformin, provided that renal function is normal and neither B_12_ deficiency nor polyneuropathy are present^9^. By adding other antidiabetic agents to metformin, improved control of glycated haemoglobin (HbA_1_c) and blood glucose levels is achieved^11^. In various countries the use of metformin and dipeptidyl peptidase IV inhibitors has increased^12–14^, whereas the use of sulfonylureas, glitazones, and α-glucosidase inhibitors has decreased^15–17^. However, there is insufficient evidence about how drug prescriptions have changed over time in Switzerland.

We thus aimed to assess the changes in diabetes management and antidiabetic drug administration in French-speaking Switzerland. Our hypothesis was that diabetes control has improved and that the number of antidiabetic drugs available and used in clinical practice has increased over the years.

## Materials and methods

### Participants

The Bus Santé study conducts annual health examination surveys since 1992 among circa 1000 men and women drawn from independent samples of residents aged 35–74 living in the state of Geneva, Switzerland^18^. The random selection in age and gender strata was proportional to the corresponding frequencies in the population. A first invitation letter was sent to a potential respondent; in the case of a non-response, up to seven telephone attempts were made to reach the person at different times of the day and on different days of the week, including Saturday and Sunday. If a selected person could not be reached by telephone, two further mailings were sent. One person who had not been reached after three mailings and seven phone calls was replaced following the same selection protocol. The recruitment of a potential subject took between 2 and 2 months^19^.

### Diabetes information

Fasting plasma blood samples were collected and glucose levels were assayed using commercially available enzymatic kits (Bayer Technicon Diagnostics, CV 1.4%).

Diagnosed diabetes was defined as the participants reporting themselves as being diagnosed with diabetes. Diabetes prevalence was defined as a fasting plasma glucose level ≥ 7 mmol/L and/or being diagnosed with diabetes. Treated diabetes was defined among participants diagnosed with diabetes as the presence of any antidiabetic drug. Controlled diabetes was defined among participants treated for diabetes as a fasting plasma glucose level < 6.7 mmol/L. Antidiabetic drugs were self-reported and categorized into sulfonylureas, biguanides, insulin, others [thiazolidines, sulphonamides, sodium-glucose cotransporter-2 (SGLT-2) inhibitors, dipeptidyl peptidase 4 (DPP4) inhibitors and glucagon-like peptide 1 (GLP-1) agonists] and combinations (medicines containing any two or more of the previous categories).

### Covariates

Socio-demographic data were self-reported. Nationality was categorized as Swiss and other. Marital status was categorized as single, married or in couple, divorced, and widowed. Educational level was categorized into primary, secondary, and tertiary. Smoking status was categorized as never, former (irrespective of the time since quitting) and current. Personal history of cardiovascular disease (CVD) was categorized as present/absent.

Height and weight were measured with participants in light clothes using standard procedures. Body mass index (BMI) was calculated and categorized as normal (< 25 kgm^2^), overweight (≥ 25 and < 30 kg/m^2^) and obese (≥ 30 kg/m^2^). Blood pressure (BP) was measured thrice in the sitting position on the right arm after at least 10 min rest using a standard protocol and a validated automated oscillometric sphygmomanometer. Hypertension was defined as a systolic BP ≥ 140 mm Hg and/or a diastolic BP ≥ 90 mm Hg and/or self-reported information of antihypertensive drug therapy.

### Exclusion criteria

Participants were excluded if they a) missed any data about diabetes awareness and/or antidiabetic drug medication; b) had missing information for any covariate.

### Statistical analysis

Descriptive results were presented as number of participants (percentage) for categorical variables or mean ± standard deviation for continuous variables. Bivariate analyses were performed using the chi-square test for categorical variables and the student’s t-test for continuous variables. Multivariable analysis was performed using logistic regression overall and stratified by gender, and the results were presented as odds ratio (OR) and 95% confidence interval (CI). Possible interactions between gender and BMI categories, hypertension, and history of CVD were also assessed. Statistical analyses were conducted using Stata version 17.0 for windows (Stata Corp, College Station, Texas, USA) and statistical significance was determined with *p* < 0.05 in a two-sided test.

### Ethical statement

The Bus Santé study was approved by the local institutional review board (Commission Cantonale d’Ethique de la Recherche de Genève; IRB00003116). All research was performed in accordance with the relevant guidelines and regulations. All participants provided written informed consent^20^.

## Results

### Characteristics of participants

Comparison of the factors between included and excluded participants is summarized in Supplementary Table 1. Included participants were younger, had a lower BMI, and were more likely to have dyslipidaemia compared to excluded participants. The characteristics according to gender are summarized in Table 1. Women presented with a lower educational level, were more frequently of Swiss nationality, divorced, never smokers, with normal weight, and presented less frequently with hypertension or history of CVD.

**Table 1:** Characteristics of the sample, by gender, overall and stratified by gender.

	Men	Women	*p*-value
N	5964	6384	
Study period			
2005–9	978 (16.4)	1036 (16.2)	0.721
2010–4	2360 (39.6)	2491 (39.0)	
2015–9	2626 (44.0)	2857 (44.8)	
Age (years)	48.7 ± 13.5	48.4 ± 13.4	0.212
Educational level (%)			
Primary	421 (7.1)	569 (8.9)	< 0.001
Secondary	2576 (43.2)	2870 (45.0)	
Tertiary	2967 (49.8)	2945 (46.1)	
Swiss nationality (%)	3883 (65.1)	4346 (68.1)	< 0.001
Marital status (%)			
Single	993 (16.7)	1134 (17.8)	< 0.001
Married/couple	4070 (68.2)	3929 (61.5)	
Divorced	571 (9.6)	1063 (16.7)	
Widowed	330 (5.5)	258 (4.0)	
Smoking status (%)			
Never	2627 (44.1)	3398 (53.2)	< 0.001
Former	1943 (32.6)	1712 (26.8)	
Current	1394 (23.4)	1274 (20.0)	
BMI (kg/m^2^)	25.9 ± 3.9	24.2 ± 4.9	< 0.001
BMI categories (%)			
Normal	2644 (44.3)	4178 (65.4)	< 0.001
Overweight	2474 (41.5)	1480 (23.2)	
Obese	846 (14.2)	726 (11.4)	
Hypertension (%)	1609 (27.0)	1096 (17.2)	< 0.001
Dyslipidaemia (%)	1848 (59.7)	1473 (60.7)	0.450
History of CVD (%)	327 (5.5)	183 (2.9)	< 0.001

*BMI* body mass index, *CVD* cardiovascular disease. Results are expressed as number of participants (column percentage) for categorical variables and as average ± standard deviation for continuous variables. Comparisons between genders performed using chi-square for categorical variables and student’s t-test for continuous variables.

### Trends in diabetes prevalence, awareness, treatment, and control

The trends in diabetes prevalence, awareness, treatment, and control overall and stratified by gender are summarized in Table 2. Total and diagnosed diabetes prevalence (awareness) decreased overall and in men, while no changes were found in women, and in control rates. Treatment rates did not improve overall but increased in men.

**Table 2:** Trends in diabetes prevalence, awareness, treatment and control, Bus Santé study, Geneva, Switzerland (2005–2019), overall and stratified by gender.

	Prevalence			Awareness			Treatment			Control		
	All	Men	Women	All	Men	Women	All	Men	Women	All	Men	Women
2005–9	176 (8.7)	105 (10.7)	71 (6.9)	141 (7.0)	78 (8.0)	63 (6.1)	63 (44.1)	34 (43.0)	29 (45.3)	19 (30.2)	7 (20.6)	12 (41.4)
2010–4	318 (6.6)	189 (8.0)	129 (5.2)	273 (5.6)	159 (6.7)	114 (4.6)	146 (51.8)	97 (59.5)	49 (41.2)	46 (31.5)	26 (26.8)	20 (40.8)
2015–9	341 (6.2)	189 (7.2)	152 (5.3)	284 (5.2)	147 (5.6)	137 (4.8)	153 (51.9)	100 (64.1)	53 (38.1)	52 (34.0)	28 (28.0)	24 (45.3)
*P* for trend	< 0.001	0.001	0.144	0.010	0.007	0.213	0.251	0.004	0.331	0.830	0.451	0.690

Results are expressed as number of participants and (percentage). For treatment, the denominator is the number of participants diagnosed with diabetes; for control, the denominator is the number of participants reporting being treated. Trends were assessed using logistic regression.

### Factors associated with diabetes prevalence, awareness, treatment, and control

The results of the bivariate analysis of the factors associated with diabetes prevalence, awareness, treatment, and control are summarized in Table 3. Participants with prevalent or diagnosed diabetes were less frequently women, older, had a lower educational level, were more frequently divorced and less frequently single, were more frequently former smokers, had a higher BMI and a higher frequency of obesity, hypertension, dyslipidaemia, and history of CVD.

**Table 3:** Bivariate analysis of the factors associated with diabetes prevalence, awareness, treatment and control, Bus Santé study, Geneva, Switzerland (2005–2019).

	Prevalence of diabetes		*p*-value	Diagnosis of diabetes		*p*-value
	No	Yes		No	Yes	
N	11,513	835		11,645	698	
Woman (%)	6032 (52.4)	352 (42.2)	< 0.001	6068 (52.1)	314 (45.0)	< 0.001
Age (years)	47.9 ± 13.4	57.7 ± 11.1	< 0.001	48.0 ± 13.4	57.9 ± 11.1	< 0.001
Educational level (%)						
Primary	879 (7.6)	111 (13.3)	< 0.001	894 (7.7)	96 (13.8)	< 0.001
Secondary	5034 (43.7)	412 (49.3)		5104 (43.8)	339 (48.6)	
Tertiary	5600 (48.6)	312 (37.4)		5647 (48.5)	263 (37.7)	
Swiss nationality (%)	7677 (66.7)	552 (66.1)	0.734	7755 (66.6)	471 (67.5)	0.631
Marital status (%)						
Single	2051 (17.8)	76 (9.1)	< 0.001	2065 (17.7)	61 (8.7)	< 0.001
Married/couple	7429 (64.5)	570 (68.3)		7510 (64.5)	486 (69.6)	
Divorced	1484 (12.9)	150 (18.0)		1512 (13.0)	121 (17.3)	
Widowed	549 (4.8)	39 (4.7)		558 (4.8)	30 (4.3)	
Smoking status (%)						
Never	5672 (49.3)	353 (42.3)	< 0.001	5722 (49.1)	303 (43.4)	< 0.001
Former	3343 (29.0)	312 (37.4)		3396 (29.2)	256 (36.7)	
Current	2498 (21.7)	170 (20.4)		2527 (21.7)	139 (19.9)	
BMI (kg/m^2^)	24.8 ± 4.2	28.6 ± 6.5	< 0.001	24.9 ± 4.3	28.4 ± 6.8	< 0.001
BMI categories (%)						
Normal	6590 (57.2)	232 (27.8)	< 0.001	6612 (56.8)	207 (29.7)	< 0.001
Overweight	3662 (31.8)	292 (35.0)		3707 (31.8)	246 (35.2)	
Obese	1261 (11.0)	311 (37.3)		1326 (11.4)	245 (35.1)	
Hypertension (%)	2281 (19.8)	424 (50.8)	< 0.001	2349 (20.2)	355 (50.9)	< 0.001
Dyslipidaemia (%)	2844 (57.4)	477 (84.4)	< 0.001	2887 (57.4)	433 (88.9)	< 0.001
History of CVD (%)	399 (3.5)	111 (13.3)	< 0.001	415 (3.6)	94 (13.5)	< 0.001

	Treatment of diabetes §		*p*-value	Control of diabetes †		*p*-value
	No	Yes		No	Yes	
N	358	362		245	117	
Woman (%)	191 (53.4)	131 (36.2)	< 0.001	75 (30.6)	56 (47.9)	0.001
Age (years)	54.4 ± 11.8	61.1 ± 9.4	< 0.001	61.5 ± 8.7	60.4 ± 10.8	0.294
Educational level (%)						
Primary	41 (11.5)	62 (17.1)	0.034	41 (16.7)	21 (18.0)	0.960
Secondary	170 (47.5)	178 (49.2)		121 (49.4)	57 (48.7)	
Tertiary	147 (41.1)	122 (33.7)		83 (33.9)	39 (33.3)	
Swiss nationality (%)	242 (67.6)	242 (66.9)	0.831	164 (66.9)	78 (66.7)	0.959
Marital status (%)						
Single	37 (10.3)	27 (7.5)	0.501	16 (6.5)	11 (9.4)	0.265
Married/couple	244 (68.2)	256 (70.7)		181 (73.9)	75 (64.1)	
Divorced	63 (17.6)	61 (16.9)		38 (15.5)	23 (19.7)	
Widowed	14 (3.9)	18 (5.0)		10 (4.1)	8 (6.8)	
Smoking status (%)						
Never	159 (44.4)	155 (42.8)	0.803	100 (40.8)	55 (47.0)	0.179
Former	126 (35.2)	136 (37.6)		100 (40.8)	36 (30.8)	
Current	73 (20.4)	71 (19.6)		45 (18.4)	26 (22.2)	
BMI (kg/m^2^)	26.6 ± 4.8	30.1 ± 7.8	< 0.001	29.9 ± 4.9	30.4 ± 11.8	0.526
BMI categories (%)						
Normal	141 (39.4)	73 (20.2)	< 0.001	39 (15.9)	34 (29.1)	0.014
Overweight	141 (39.4)	118 (32.6)		83 (33.9)	35 (29.9)	
Obese	76 (21.2)	171 (47.2)		123 (50.2)	48 (41.0)	
Hypertension (%)	125 (34.9)	238 (65.8)	< 0.001	161 (65.7)	77 (65.8)	0.985
Dyslipidaemia (%)	192 (86.1)	253 (91.0)	0.083	171 (90.5)	82 (92.1)	0.652
History of CVD (%)	27 (7.5)	67 (18.5)	< 0.001	44 (18.0)	23 (19.7)	0.697

*BMI* body mass index, *CVD* cardiovascular disease. Results are expressed as number of participants (column percentage) for categorical variables and as average ± standard deviation for continuous variables. Comparisons between groups within each status (prevalence, diagnosis, treatment, and control) performed using chi-square for categorical variables and student’s t-test for continuous variables.

§, among participants diagnosed with diabetes; †, among participants treated for diabetes.

Among participants diagnosed with diabetes, participants reporting being treated were less frequently women, were older, had a lower educational level, a higher BMI and a higher frequency of obesity, hypertension, and history of CVD (Table 3).

Among participants treated for diabetes, participants achieving adequate control were more frequently women and less frequently obese, while no significant difference was found for the other covariates (Table 3).

The results of the multivariable analysis of the factors associated with diabetes prevalence, awareness, treatment, and control are summarized in Table 4. Being a woman, increased educational level and being a Swiss national were negatively associated, while increased age, increased BMI, smoking, presence of hypertension or personal history of CVD were positively associated with diabetes prevalence. Similar findings were observed for diagnosis of diabetes, although being a woman, Swiss nationality or smoking status were no longer statistically relevant.

**Table 4:** Multivariable analysis of the factors associated with diabetes prevalence, awareness, treatment and control, Bus Santé study, Geneva, Switzerland (2005–2019).

	Prevalence	*p*–value	Diagnosis	*p*–value	Treatment §	*p*–value	Control †	*p*–value
N	12,348		12,343		720		362	
Period								
2005–9	1 (ref.)		1 (ref.)		1 (ref.)		1 (ref.)	
2010–4	0.78 (0.64–0.95)	0.016	0.85 (0.68–1.06)	0.150	1.57 (1.00–2.49)	0.052	1.28 (0.64–2.54)	0.487
2015–9	0.88 (0.72–1.07)	0.203	0.93 (0.75–1.15)	0.501	1.84 (1.17–2.91)	0.009	1.36 (0.68–2.68)	0.383
P-value for trend	0.203		0.501		0.009		0.383	
Woman vs. man	0.81 (0.69–0.95)	0.009	0.93 (0.78–1.10)	0.378	0.65 (0.45–0.92)	0.016	2.19 (1.30–3.69)	0.003
Age (per decade)	1.53 (1.43–1.65)	< 0.001	1.53 (1.42–1.65)	< 0.001	1.50 (1.26–1.79)	< 0.001	0.91 (0.69–1.19)	0.482
Educational level (%)								
Primary	1 (ref.)		1 (ref.)		1 (ref.)		1 (ref.)	
Secondary	0.84 (0.66–1.06)	0.145	0.78 (0.60–1.01)	0.058	0.70 (0.42–1.18)	0.179	0.84 (0.43–1.65)	0.615
Tertiary	0.76 (0.59–0.98)	0.033	0.73 (0.56–0.95)	0.021	0.66 (0.39–1.14)	0.135	0.88 (0.42–1.82)	0.725
*P*-value for trend	0.033		0.021		0.135		0.725	
Swiss nationality versus other	0.82 (0.70–0.97)	0.021	0.89 (0.74–1.06)	0.194	0.91 (0.63–1.32)	0.621	1.02 (0.61–1.71)	0.940
Marital status								
Single	1 (ref.)		1 (ref.)		1 (ref.)		1 (ref.)	
Married/couple	1.06 (0.82–1.37)	0.674	1.15 (0.86–1.52)	0.347	1.45 (0.80–2.61)	0.223	0.59 (0.25–1.43)	0.244
Divorced	1.33 (0.98–1.81)	0.063	1.31 (0.94–1.82)	0.113	1.70 (0.85–3.39)	0.132	0.78 (0.29–2.11)	0.621
Widowed	0.93 (0.61–1.41)	0.732	0.91 (0.57–1.44)	0.684	1.29 (0.50–3.33)	0.603	1.90 (0.53–6.81)	0.324
Smoking status								
Never	1 (ref.)		1 (ref.)		1 (ref.)		1 (ref.)	
Former	1.13 (0.95–1.34)	0.165	1.09 (0.91–1.30)	0.372	0.75 (0.51–1.10)	0.139	0.87 (0.50–1.51)	0.611
Current	1.26 (1.03–1.54)	0.027	1.21 (0.98–1.51)	0.083	1.15 (0.73–1.80)	0.551	1.10 (0.59–2.07)	0.763
*P*-value for trend	0.027		0.083		0.551		0.763	
BMI categories (%)								
Normal	1 (ref.)		1 (ref.)		1 (ref.)		1 (ref.)	
Overweight	1.54 (1.28–1.86)	< 0.001	1.48 (1.21–1.80)	< 0.001	1.18 (0.78–1.80)	0.435	0.47 (0.24–0.89)	0.021
Obese	4.21 (3.46–5.13)	< 0.001	3.50 (2.83–4.33)	< 0.001	2.89 (1.85–4.52)	< 0.001	0.38 (0.20–0.73)	0.003
*P*-value for trend	< 0.001		< 0.001		< 0.001		0.003	
Hypertension (yes vs. no)	1.60 (1.35–1.90)	< 0.001	1.65 (1.37–1.98)	< 0.001	1.94 (1.33–2.83)	0.001	1.50 (0.86–2.60)	0.152
History of CVD (yes vs. no)	2.05 (1.60–2.61)	< 0.001	2.05 (1.58–2.65)	< 0.001	1.69 (1.00–2.87)	0.049	1.46 (0.79–2.68)	0.227

BMI, body mass index; CVD, cardiovascular disease. §, among participants diagnosed with diabetes; †, among participants treated for diabetes. Results are expressed as odds ratio and (95% confidence interval). Statistical analysis by logistic regression.

Being a woman was negatively associated with treatment of diabetes. Increased age, obese BMI, presence of hypertension or personal history of CVD were positively associated with treatment of diabetes. Being a woman was positively associated with diabetes control, while increased BMI was negatively associated with diabetes control.

Comparable findings were obtained when the analysis was stratified by gender (Supplementary Tables 2 and 3 for men and women, respectively), although several associations were no longer significant. Interaction analysis showed that women with hypertension had a higher likelihood of presenting with diabetes, being diagnosed, treated, and controlled than men, while men with history of CVD had a higher likelihood of presenting with diabetes than women; no significant interaction was found for BMI categories.

Multivariable analysis including dyslipidaemia is summarized in Supplementary Table 4. Presence of dyslipidaemia was positively associated with diabetes prevalence and awareness. After adjusting for dyslipidaemia, the association between diabetes prevalence and increased educational level or smoking was no longer statistically significant. After adjusting for dyslipidaemia, being a woman was additionally negatively associated with diagnosis of diabetes, while the association with educational level was no longer statistically significant. After adjusting for dyslipidaemia, divorced marital status was additionally positively associated with treatment of diabetes, while the association with presence of personal history of CVD was no longer statistically significant. After adjusting for dyslipidaemia, diabetes control remained positively associated with women gender and negatively associated with increased BMI.

### Antidiabetic drugs

**Figure 1:**
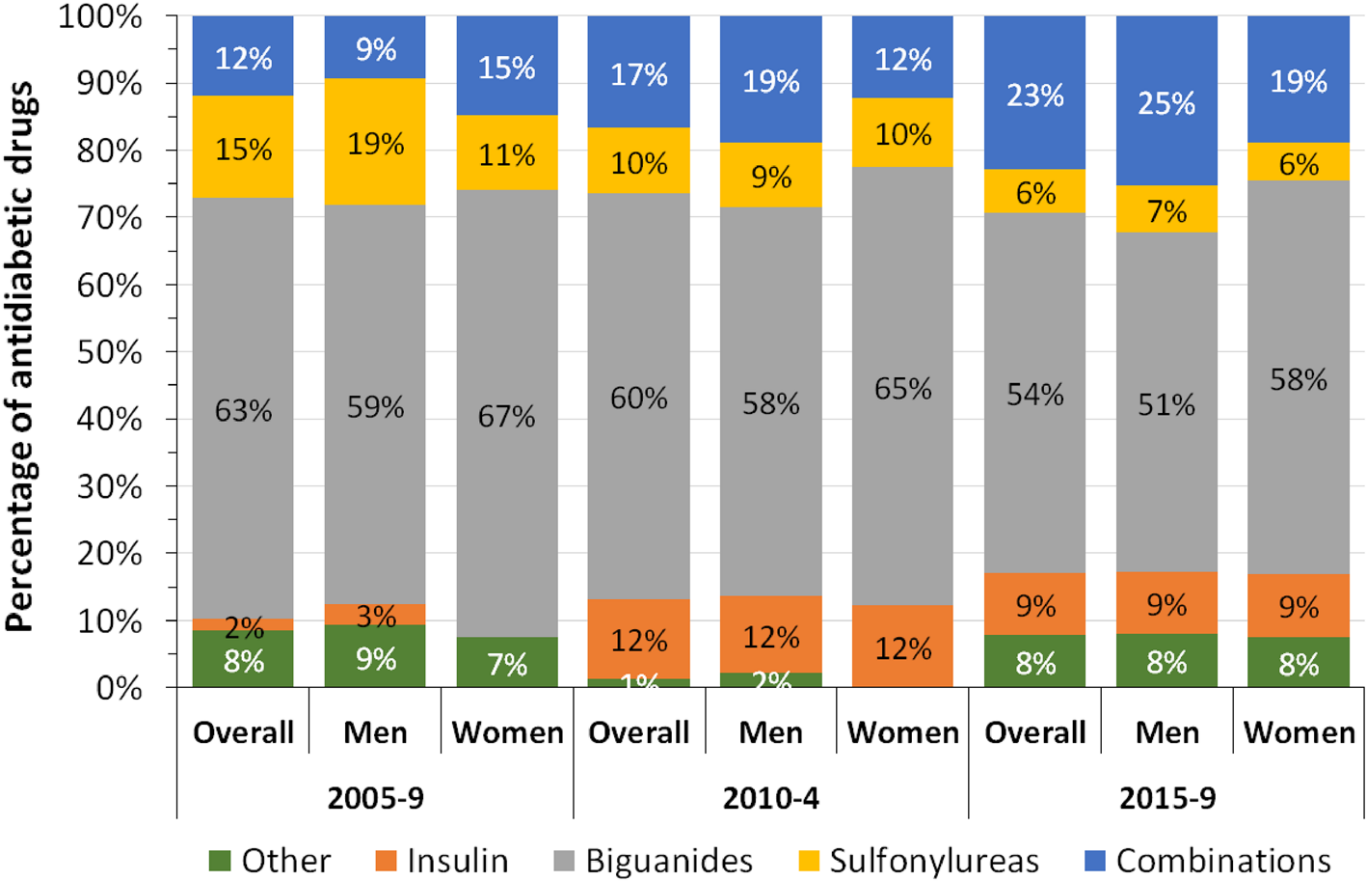
Percentage of antidiabetic drugs, Bus Santé study, Geneva, Switzerland (2005–2019), overall and stratified by gender.

Trends in antidiabetic drugs expressed as percentage of all antidiabetic drugs are summarized overall and for each gender in Fig. 1. Percentage of antidiabetic combinations increased from 12% in 2005–9 to 23% in 2015–9. Percentage of sulfonylureas and biguanides decreased between 2005–9 and 2015–9 (15–6% for sulfonylureas and 63–54% for biguanides). Trends for insulin and other drugs were inconsistent. Similar findings were observed when the analysis was stratified by gender.

## Discussion

This study aimed to assess diabetes prevalence, diagnosis, treatment, and control in the adult population of French-speaking Switzerland. Our results suggest that treatment and control rates among people with diabetes remain low and that the combination of different antidiabetic drugs is increasingly used in the treatment of diabetes.

### Diabetes prevalence and awareness

Our results suggest that overall diabetes prevalence and awareness have decreased. Our findings do not replicate the global increasing trends in diabetes prevalence and incidence of the last decades^1^. Nevertheless, our findings are in agreement with data from some high-income countries, where diabetes incidence has been stable or decreasing for the last years^21^. A possible explanation could be the increase in physical activity levels of the Swiss population, as in 2017, almost three-quarters of the population complied with physical activity recommendations, a 14% increase from 2002^22^. Still, a recruitment bias cannot be excluded, through participation of more health-conscious people. It would thus be important to confirm our findings in other population-based surveys.

Dyslipidaemia and hypertension were positively associated with prevalence and diagnosis of diabetes. Those findings show that CVD risk factors tend to cluster among diabetic patients^23^, and that presence of one risk factor prompts the physician to search for the other ones.

### Treatment and control of diabetes

Among participants diagnosed with diabetes, treatment and control rates did not change. Only one-third (34%) of treated participants was controlled, a lower rate than in a study conducted in 16 European countries, where 65.2% achieved the HbA_1_c target of < 7.0%^4^. The difference could be partly explained by the different definitions and thresholds for controlled diabetes (target of fasting plasma glucose level < 6.7 mmol/L in our study vs. HbA_1_c < 7.0% in the European study)^24^. The reasons for such a low control rate could be due to clinical inertia, participants with diabetes and their doctors not complying with treatment or not willing to intensify it^25,26^. It would be important that future studies assess compliance to treatment, by for example studying drug prescriptions.

After multivariable analysis, women with diabetes were less likely to be treated but more likely to be controlled. Findings of previous studies on gender differences in the treatment and control of diabetes are inconsistent. Several studies conducted in Iran, China and the EU reported that both genders were equally treated for diabetes^27–29^. Regarding control levels, an Iranian study reported that women were more likely to achieve diabetes control^27^; the opposite was reported by a Chinese and an European study^28,29^, while a Pakistani study reported that both genders had similar control levels^30^. The reasons for a lower treatment rate among women diagnosed with diabetes are currently unknown and should be further assessed. Possible explanations include the underestimation of diabetes severity in women, lack of staff for patient education, doctors’ lack of updated knowledge, or patients’ lack of willingness to be treated^31,32^.

After multivariable analysis, obese participants with diabetes were more likely to be treated but less likely to be controlled. Those findings are in agreement with a previous study, where high BMI was a strong predictor of receiving antidiabetic treatment^33^. As obesity is frequently associated with concomitant diseases such as cardiovascular diseases and cancer^34^, doctors might feel more compelled to prescribe treatment. However, treatment of obese patients with diabetes is complicated by metabolic and drug interactions that could prevent glycaemic control^35^.

### Trends in antidiabetic drugs

Use of antidiabetic combinations increased, while use of sulfonylureas and biguanides decreased. The trend of combination therapy has not been studied frequently, but increased use has been noted previously^15^. Sulfonylureas and biguanides are the oldest noninsulin injectable antidiabetic agents^36^. In previous studies, use of sulfonylureas decreased similarly, but contrary to our results, use of metformin (biguanide) increased^13,15,17^.

In our study, no trend was found for insulin and other drugs. Contrary to our results, insulin use increased in several other studies^12,13,15,17^. The European Medicine Agency and the US Food and Drug Administration authorized use of three SGLT-2 inhibitors (dapagliflozin, canagliflozin, and empagliflozin) between 2012 and 2015^37^. The first GLP-1 receptor agonists exenatide was approved in 2005 (US) and 2006 (Europe)^38^. Although some drugs were relatively new, others could have been prescribed for almost 10 years as it is the case for GLP-1 agonists, or for five years for some SGLT-2 inhibitors. Overall, our results suggest that physicians in Geneva appear to be reluctant to prescribe the newest antidiabetic drugs. Whether such attitude is due to clinical inertia or to a constraint by health insurances (as the newest antidiabetic drugs are more expensive and thus less likely to be reimbursed) remains to be assessed.

### Clinical implications

Our results suggest that certain subgroups of individuals with diabetes are more likely to be treated or to achieve diabetes control. Particular attention should be paid to patients with high BMI, who are more likely to have long-term hyperglycaemia and are therefore at higher risk of adverse diabetes-related complications. Efforts to increase diabetes control should be implemented among patients and their physicians, as inadequately controlled diabetes leads to increased health and economic costs^39,40^.

Combination therapy progressively replaced sulfonylureas and biguanides. Newer antidiabetic drugs such as SGLT-2 inhibitors and GLP-1 receptor agonists were less prescribed. These drugs reduce cardiovascular and renal damage and could be of great benefit to participants with diabetes^41^. Hence, it would be important that doctors be made aware of these benefits, and that health insurers accept to reimburse the drugs^42^.

### Strengths and limitations

This study has several strengths. Firstly, it assessed four important parameters of diabetes management in Switzerland (prevalence, awareness, treatment, and control), and at three different time points. Secondly, it assessed the parameters’ association with a broad spectrum of socio-demographic variables allowing better understanding of the factors that hinder or promote optimal diabetes management.

This study also has several limitations. Firstly, it was conducted in a single location, so the results may not be transferable to other environments. Secondly, antidiabetic drugs were self-reported. Self-report has overestimated medication adherence in the past^43^. Consequently, the overall results might be overoptimistic, and the true status of antidiabetic drug use might actually be worse than reported. Still, self-reporting should not affect the differences between groups and associations with different factors. Thirdly, the sample size might not be sufficient to detect minor differences in the association of the factors with diabetes prevalence, awareness, treatment, and control. Still, such small differences could be clinically irrelevant. Fourthly, no information regarding history of childbearing or gestational diabetes was available for women; hence, we could not adjust for those potential confounders. Finally, the sample size resulted low when adjusting for dyslipidaemia because participants lacked data for dyslipidaemia status. Still, only few of the significant results changed after multivariable analysis leaving out dyslipidaemia status.

## Conclusion

In canton Geneva, trends in drug prescription are changing, with combination therapy gaining importance. However, only about half of participants diagnosed with diabetes receive antidiabetic treatment, and only one third of participants treated for diabetes are controlled, with no significant improvements in the last 10 years.

### Supplementary Information

Supplementary Tables.

## Data Availability

Due to the sensitivity of the data and the lack of consent for online posting, individual data cannot be made accessible. Non-identifiable, individual-level data are available for interested researchers, who meet the criteria for access to confidential data sharing. Requests to access the data should be directed to Professor Idris Guessous at Idris.Guessous@hcuge.ch.
