# Supplementary material for: Trends in diabetes prevalence, awareness, treatment, and control in French-speaking Switzerland
Source: Sci Rep. 2024 Feb 28;14:4839. doi: 10.1038/s41598-024-54856-6 (PMC10902360; doi:10.1038/s41598-024-54856-6)
Supplement: Supplementary file 1 — Supplementary Tables. [file 41598_2024_54856_MOESM1_ESM.docx]

**Supplementary information**

**Supplementary table 1:** comparison of the factors between included and excluded participants, Bus Santé study, Geneva, Switzerland.

|  | **Included** | **Excluded** | **p--value** |
| --- | --- | --- | --- |
| **N** | **12,348** | **varies** |  |
| Woman (%) | 6384 (51.7) | 382 (49.8) | 0.308 |
| Age (years) | 48.6 ± 13.5 | 50.3 ± 13.8 | <0.001 |
| Educational level (%) |  |  | 0.782 |
| Primary | 990 (8.0) | 53 (8.6) |  |
| Secondary | 5446 (44.1) | 275 (44.7) |  |
| Tertiary | 5912 (47.9) | 287 (46.7) |  |
| Swiss nationality (%) | 8229 (66.6) | 531 (68.5) | 0.283 |
| Marital status (%) |  |  | 0.575 |
| Single | 2127 (17.2) | 124 (16.1) |  |
| Married/couple | 7999 (64.8) | 493 (64.1) |  |
| Divorced | 1634 (13.2) | 114 (14.8) |  |
| Widowed | 588 (4.8) | 38 (4.9) |  |
| Smoking status (%) |  |  | 0.943 |
| Never | 6025 (48.8) | 343 (48.4) |  |
| Former | 3655 (29.6) | 209 (29.5) |  |
| Current | 2668 (21.6) | 157 (22.1) |  |
| BMI (kg/m^2^) | 25.1 ± 4.5 | 25.5 ± 5.0 | 0.020 |
| BMI categories (%) |  |  | 0.044 |
| Normal | 6822 (55.3) | 254 (51.5) |  |
| Overweight | 3954 (32.0) | 158 (32.1) |  |
| Obese | 1572 (12.7) | 81 (16.4) |  |
| Hypertension (%) | 2705 (21.9) | 168 (21.7) | 0.881 |
| Dyslipidaemia (%) | 3321 (60.1) | 219 (34.8) | <0.001 |
| History of CVD (%) | 510 (4.1) | 38 (4.9) | 0.297 |

BMI, body mass index; CVD, cardiovascular disease. Results are expressed as number of participants (column percentage) for categorical variables and as average ± standard deviation for continuous variables. Comparisons performed using chi-square for categorical variables and student’s t-test for continuous variables.

**Supplementary table 2:** Multivariable analysis of the factors associated with diabetes prevalence, awareness, treatment and control in men, Bus Santé study, Geneva, Switzerland (2005-2019).

|  | **Prevalence** | **p--value** | **Diagnosis** | **p--value** | **Treatment §** | **p--value** | **Control †** | **p--value** |
| --- | --- | --- | --- | --- | --- | --- | --- | --- |
| **N** | **5,964** |  | **5,961** |  | **398** |  | **231** |  |
| Period |  |  |  |  |  |  |  |  |
| 2005-9 | 1 (ref.) |  | 1 (ref.) |  | 1 (ref.) |  | 1 (ref.) |  |
| 2010-4 | 0.75 (0.57 - 0.99) | 0.039 | 0.88 (0.65 - 1.19) | 0.395 | 2.13 (1.17 - 3.88) | 0.013 | 1.56 (0.58 - 4.19) | 0.381 |
| 2015-9 | 0.79 (0.60 - 1.04) | 0.096 | 0.85 (0.63 - 1.15) | 0.294 | 2.97 (1.60 - 5.49) | 0.001 | 1.58 (0.59 - 4.26) | 0.363 |
| P-value for trend | 0.097 |  | 0.294 |  | <0.001 |  | 0.363 |  |
| Age (per decade) | 1.85 (1.67 - 2.04) | <0.001 | 1.93 (1.72 - 2.16) | <0.001 | 1.52 (1.18 - 1.96) | 0.001 | 0.80 (0.54 - 1.17) | 0.252 |
| Educational level (%) |  |  |  |  |  |  |  |  |
| Primary | 1 (ref.) |  | 1 (ref.) |  | 1 (ref.) |  | 1 (ref.) |  |
| Secondary | 0.73 (0.52 - 1.02) | 0.068 | 0.66 (0.46 - 0.97) | 0.032 | 0.63 (0.30 - 1.31) | 0.214 | 0.91 (0.34 - 2.47) | 0.858 |
| Tertiary | 0.64 (0.45 - 0.90) | 0.012 | 0.62 (0.42 - 0.91) | 0.014 | 0.70 (0.33 - 1.47) | 0.346 | 0.79 (0.29 - 2.20) | 0.658 |
| P-value for trend | 0.012 |  | 0.014 |  | 0.346 |  | 0.658 |  |
| Swiss nationality vs. other | 0.79 (0.64 - 0.99) | 0.038 | 0.89 (0.70 - 1.14) | 0.355 | 1.09 (0.67 - 1.77) | 0.727 | 1.10 (0.55 - 2.20) | 0.785 |
| Marital status |  |  |  |  |  |  |  |  |
| Single | 1 (ref.) |  | 1 (ref.) |  | 1 (ref.) |  | 1 (ref.) |  |
| Married/couple | 0.75 (0.53 - 1.08) | 0.119 | 0.72 (0.49 - 1.07) | 0.102 | 1.53 (0.71 - 3.28) | 0.273 | 1.84 (0.47 - 7.17) | 0.379 |
| Divorced | 1.07 (0.70 - 1.65) | 0.750 | 0.87 (0.54 - 1.41) | 0.576 | 2.04 (0.79 - 5.25) | 0.141 | 2.49 (0.52 - 11.9) | 0.253 |
| Widowed | 0.73 (0.43 - 1.24) | 0.245 | 0.71 (0.39 - 1.26) | 0.240 | 1.49 (0.47 - 4.70) | 0.501 | 3.31 (0.61 - 18.1) | 0.167 |
| Smoking status |  |  |  |  |  |  |  |  |
| Never | 1 (ref.) |  | 1 (ref.) |  | 1 (ref.) |  | 1 (ref.) |  |
| Former | 1.22 (0.97 - 1.53) | 0.097 | 1.17 (0.91 - 1.51) | 0.215 | 0.98 (0.59 - 1.63) | 0.952 | 0.81 (0.4 - 1.64) | 0.550 |
| Current | 1.37 (1.03 - 1.81) | 0.028 | 1.33 (0.97 - 1.81) | 0.073 | 1.30 (0.71 - 2.40) | 0.392 | 0.98 (0.42 - 2.27) | 0.961 |
| P-value for trend | 0.027 |  | 0.073 |  | 0.393 |  | 0.961 |  |
| BMI categories (%) |  |  |  |  |  |  |  |  |
| Normal | 1 (ref.) |  | 1 (ref.) |  | 1 (ref.) |  | 1 (ref.) |  |
| Overweight | 1.30 (1.00 - 1.69) | 0.046 | 1.24 (0.93 - 1.65) | 0.140 | 1.26 (0.70 - 2.25) | 0.437 | 0.31 (0.13 - 0.75) | 0.009 |
| Obese | 4.17 (3.16 - 5.51) | <0.001 | 3.43 (2.53 - 4.65) | <0.001 | 2.84 (1.51 - 5.33) | 0.001 | 0.37 (0.16 - 0.86) | 0.020 |
| P-value for trend | <0.001 |  | <0.001 |  | 0.001 |  | 0.020 |  |
| Hypertension (yes vs. no) | 1.57 (1.25 - 1.97) | <0.001 | 1.58 (1.23 - 2.03) | <0.001 | 1.45 (0.89 - 2.37) | 0.135 | 0.95 (0.47 - 1.91) | 0.880 |
| History of CVD (yes vs. no) | 2.19 (1.63 - 2.95) | <0.001 | 2.13 (1.56 - 2.90) | <0.001 | 2.21 (1.17 - 4.16) | 0.014 | 1.69 (0.83 - 3.46) | 0.151 |

BMI, body mass index; CVD, cardiovascular disease. §, among participants diagnosed with diabetes; †, among participants treated for diabetes. Results are expressed as odds ratio and (95% confidence interval). Statistical analysis by logistic regression.

**Supplementary table 3:** Multivariable analysis of the factors associated with diabetes prevalence, awareness, treatment and control in women, Bus Santé study, Geneva, Switzerland (2005-2019).

|  | **Prevalence** | **p--value** | **Diagnosis** | **p--value** | **Treatment §** | **p--value** | **Control †** | **p--value** |
| --- | --- | --- | --- | --- | --- | --- | --- | --- |
| **N** | **6,384** |  | **6,382** |  | **322** |  | **131** |  |
| Period |  |  |  |  |  |  |  |  |
| 2005-9 | 1 (ref.) |  | 1 (ref.) |  | 1 (ref.) |  | 1 (ref.) |  |
| 2010-4 | 0.78 (0.57 - 1.07) | 0.119 | 0.78 (0.57 - 1.08) | 0.140 | 0.95 (0.45 - 1.98) | 0.884 | 1.28 (0.42 - 3.85) | 0.664 |
| 2015-9 | 0.95 (0.70 - 1.29) | 0.760 | 0.97 (0.71 - 1.34) | 0.866 | 0.95 (0.46 - 1.95) | 0.880 | 1.48 (0.48 - 4.60) | 0.496 |
| P-value for trend | 0.760 |  | 0.866 |  | 0.880 |  | 0.496 |  |
| Age (per decade) | 1.26 (1.14 - 1.40) | <0.001 | 1.23 (1.10 - 1.37) | <0.001 | 1.39 (1.08 - 1.80) | 0.012 | 0.87 (0.55 - 1.37) | 0.541 |
| Educational level (%) |  |  |  |  |  |  |  |  |
| Primary | 1 (ref.) |  | 1 (ref.) |  | 1 (ref.) |  | 1 (ref.) |  |
| Secondary | 0.91 (0.64 - 1.29) | 0.598 | 0.86 (0.60 - 1.23) | 0.417 | 0.78 (0.37 - 1.65) | 0.512 | 0.57 (0.20 - 1.66) | 0.305 |
| Tertiary | 0.86 (0.60 - 1.25) | 0.431 | 0.80 (0.54 - 1.17) | 0.246 | 0.54 (0.24 - 1.22) | 0.140 | 0.78 (0.23 - 2.69) | 0.696 |
| P-value for trend | 0.431 |  | 0.246 |  | 0.140 |  | 0.696 |  |
| Swiss nationality vs. other | 0.90 (0.70 - 1.16) | 0.429 | 0.92 (0.70 - 1.20) | 0.539 | 0.64 (0.36 - 1.16) | 0.142 | 1.02 (0.42 - 2.50) | 0.961 |
| Marital status |  |  |  |  |  |  |  |  |
| Single | 1 (ref.) |  | 1 (ref.) |  | 1 (ref.) |  | 1 (ref.) |  |
| Married/couple | 1.45 (0.98 - 2.15) | 0.060 | 1.75 (1.14 - 2.70) | 0.011 | 1.31 (0.49 - 3.49) | 0.590 | 0.07 (0.01 - 0.66) | 0.02 |
| Divorced | 1.73 (1.12 - 2.69) | 0.014 | 2.02 (1.24 - 3.27) | 0.004 | 1.53 (0.52 - 4.54) | 0.441 | 0.12 (0.01 - 1.19) | 0.07 |
| Widowed | 1.12 (0.56 - 2.24) | 0.758 | 0.96 (0.42 - 2.18) | 0.915 | 0.84 (0.13 - 5.66) | 0.860 | NC |  |
| Smoking status |  |  |  |  |  |  |  |  |
| Never | 1 (ref.) |  | 1 (ref.) |  | 1 (ref.) |  | 1 (ref.) |  |
| Former | 0.98 (0.75 - 1.28) | 0.900 | 0.96 (0.72 - 1.26) | 0.753 | 0.49 (0.26 - 0.93) | 0.029 | 1.10 (0.38 - 3.20) | 0.865 |
| Current | 1.16 (0.85 - 1.56) | 0.347 | 1.14 (0.83 - 1.57) | 0.404 | 1.05 (0.52 - 2.10) | 0.895 | 1.05 (0.35 - 3.15) | 0.929 |
| P-value for trend | 0.347 |  | 0.405 |  | 0.895 |  | 0.929 |  |
| BMI categories (%) |  |  |  |  |  |  |  |  |
| Normal | 1 (ref.) |  | 1 (ref.) |  | 1 (ref.) |  | 1 (ref.) |  |
| Overweight | 1.96 (1.49 - 2.57) | <0.001 | 1.85 (1.40 - 2.46) | <0.001 | 1.02 (0.53 - 1.96) | 0.959 | 1.10 (0.38 - 3.15) | 0.866 |
| Obese | 4.33 (3.25 - 5.77) | <0.001 | 3.58 (2.64 - 4.86) | <0.001 | 3.09 (1.58 - 6.03) | 0.001 | 0.38 (0.12 - 1.14) | 0.084 |
| P-value for trend | <0.001 |  | <0.001 |  | <0.001 |  | 0.084 |  |
| Hypertension (yes vs. no) | 1.62 (1.24 - 2.12) | <0.001 | 1.72 (1.30 - 2.28) | <0.001 | 2.96 (1.56 - 5.62) | 0.001 | 3.59 (1.25 - 10.3) | 0.017 |
| History of CVD (yes vs. no) | 1.53 (0.95 - 2.45) | 0.077 | 1.60 (0.98 - 2.61) | 0.058 | 0.75 (0.25 - 2.19) | 0.593 | 0.90 (0.21 - 3.86) | 0.885 |

BMI, body mass index; CVD, cardiovascular disease. §, among participants diagnosed with diabetes; †, among participants treated for diabetes; NC, not possible to be calculated. Results are expressed as odds ratio and (95% confidence interval). Statistical analysis by logistic regression.

**Supplementary table 4:** Multivariable analysis of the factors associated with diabetes prevalence, awareness, treatment and control, Bus Santé study, Geneva, Switzerland. Analysis including dyslipidaemia status.

|  | **Prevalence** | **p-value** | **Diagnosis** | **p-value** | **Treatment** | **p-value** | **Control** | **p-value** |
| --- | --- | --- | --- | --- | --- | --- | --- | --- |
| **N** | **5523** |  | **5521** |  | **501** |  | **278** |  |
| Period |  |  |  |  |  |  |  |  |
| 2005-9 | 1 (reference) |  | 1 (reference) |  | 1 (reference) |  | 1 (reference) |  |
| 2010-4 | 0.73 (0.56 - 0.94) | 0.015 | 0.75 (0.57 - 0.98) | 0.036 | 1.38 (0.81 - 2.36) | 0.236 | 0.93 (0.43 - 2.01) | 0.858 |
| 2015-9 | 0.89 (0.69 - 1.15) | 0.393 | 0.88 (0.67 - 1.15) | 0.347 | 1.64 (0.96 - 2.81) | 0.071 | 1.08 (0.51 - 2.31) | 0.834 |
| P-value for trend | 0.393 |  | 0.347 |  | 0.071 |  | 0.834 |  |
| Woman vs. man | 0.65 (0.53 - 0.80) | <0.001 | 0.69 (0.55 - 0.86) | 0.001 | 0.64 (0.42 - 0.99) | 0.047 | 2.22 (1.20 - 4.12) | 0.011 |
| Age (per decade) | 1.56 (1.41 - 1.71) | <0.001 | 1.53 (1.38 - 1.70) | <0.001 | 1.72 (1.36 - 2.18) | <0.001 | 0.88 (0.61 - 1.26) | 0.485 |
| Educational level (%) |  |  |  |  |  |  |  |  |
| Primary | 1 (reference) |  | 1 (reference) |  | 1 (reference) |  | 1 (reference) |  |
| Secondary | 0.83 (0.62 - 1.13) | 0.239 | 0.74 (0.54 - 1.02) | 0.064 | 0.84 (0.45 - 1.54) | 0.567 | 0.95 (0.43 - 2.11) | 0.896 |
| Tertiary | 0.76 (0.56 - 1.05) | 0.092 | 0.73 (0.53 - 1.01) | 0.059 | 0.74 (0.39 - 1.40) | 0.359 | 0.97 (0.41 - 2.27) | 0.939 |
| P-value for trend | 0.092 |  | 0.059 |  | 0.359 |  | 0.939 |  |
| Swiss nationality vs. other | 0.81 (0.65 - 0.99) | 0.047 | 0.83 (0.67 - 1.04) | 0.107 | 0.81 (0.51 - 1.27) | 0.358 | 1.06 (0.58 - 1.95) | 0.843 |
| Marital status |  |  |  |  |  |  |  |  |
| Single | 1 (reference) |  | 1 (reference) |  | 1 (reference) |  | 1 (reference) |  |
| Married/couple | 0.94 (0.67 - 1.31) | 0.702 | 1.00 (0.70 - 1.44) | 0.982 | 1.90 (0.92 - 3.95) | 0.084 | 0.78 (0.25 - 2.40) | 0.666 |
| Divorced | 1.16 (0.79 - 1.72) | 0.448 | 1.21 (0.79 - 1.84) | 0.377 | 2.66 (1.14 - 6.23) | 0.024 | 0.99 (0.28 - 3.44) | 0.982 |
| Widowed | 0.90 (0.54 - 1.51) | 0.693 | 0.92 (0.53 - 1.61) | 0.782 | 1.68 (0.55 - 5.11) | 0.362 | 2.06 (0.44 - 9.61) | 0.358 |
| Smoking status |  |  |  |  |  |  |  |  |
| Never | 1 (reference) |  | 1 (reference) |  | 1 (reference) |  | 1 (reference) |  |
| Former | 1.04 (0.84 - 1.29) | 0.722 | 1.02 (0.82 - 1.28) | 0.839 | 0.71 (0.45 - 1.12) | 0.145 | 0.90 (0.48 - 1.70) | 0.752 |
| Current | 1.26 (0.98 - 1.64) | 0.076 | 1.26 (0.96 - 1.66) | 0.095 | 1.02 (0.59 - 1.78) | 0.931 | 1.08 (0.51 - 2.27) | 0.844 |
| P-value for trend | 0.076 |  | 0.095 |  | 0.931 |  | 0.844 |  |
| BMI categories (%) |  |  |  |  |  |  |  |  |
| Normal | 1 (reference) |  | 1 (reference) |  | 1 (reference) |  | 1 (reference) |  |
| Overweight | 1.44 (1.13 - 1.83) | 0.003 | 1.37 (1.06 - 1.76) | 0.016 | 1.11 (0.67 - 1.85) | 0.675 | 0.33 (0.16 - 0.68) | 0.003 |
| Obese | 3.75 (2.91 - 4.83) | <0.001 | 3.01 (2.30 - 3.95) | <0.001 | 2.81 (1.63 - 4.84) | <0.001 | 0.28 (0.14 - 0.59) | 0.001 |
| P-value for trend | <0.001 |  | <0.001 |  | <0.001 |  | <0.001 |  |
| Hypertension (yes vs. no) | 1.45 (1.17 - 1.79) | 0.001 | 1.56 (1.24 - 1.95) | <0.001 | 1.70 (1.09 - 2.63) | 0.018 | 1.45 (0.76 - 2.74) | 0.256 |
| Dyslipidaemia (yes vs. no) | 3.13 (2.45 - 4.01) | <0.001 | 4.75 (3.52 - 6.40) | <0.001 | 1.82 (0.95 - 3.47) | 0.070 | 1.19 (0.43 - 3.27) | 0.735 |
| History of CVD (yes vs. no) | 1.58 (1.19 - 2.08) | 0.001 | 1.39 (1.04 - 1.86) | 0.028 | 1.43 (0.81 - 2.54) | 0.220 | 1.39 (0.70 - 2.77) | 0.348 |

BMI, body mass index; CVD, cardiovascular disease. Results are expressed as odds ratio and (95% confidence interval). Statistical analysis by logistic regression.
